# Effect of Layer Thickness in Selective Laser Melting on Microstructure of Al/5 wt.%Fe_2_O_3_ Powder Consolidated Parts

**DOI:** 10.1155/2014/106129

**Published:** 2014-01-02

**Authors:** Sasan Dadbakhsh, Liang Hao

**Affiliations:** ^1^College of Engineering, Mathematics and Physical Sciences, University of Exeter, Exeter EX4 4QF, UK; ^2^Department of Mechanical Engineering, KU Leuven, Celestijnenlaan 300B, P.O. Box 2420, 3001 Leuven, Belgium

## Abstract

*In situ* reaction was activated in the powder mixture of Al/5 wt.%Fe_2_O_3_ by using selective laser melting (SLM) to directly fabricate aluminium metal matrix composite parts. The microstructural characteristics of these *in situ* consolidated parts through SLM were investigated under the influence of thick powder bed, 75 **μ**m layer thickness, and 50 **μ**m layer thickness in various laser powers and scanning speeds. It was found that the layer thickness has a strong influence on microstructural outcome, mainly attributed to its impact on oxygen content of the matrix. Various microstructural features (such as granular, coralline-like, and particulate appearance) were observed depending on the layer thickness, laser power, and scanning speed. This was associated with various material combinations such as pure Al, Al-Fe intermetallics, and Al(-Fe) oxide phases formed after *in situ* reaction and laser rapid solidification. Uniformly distributed very fine particles could be consolidated in net-shape Al composite parts by using lower layer thickness, higher laser power, and lower scanning speed. The findings contribute to the new development of advanced net-shape manufacture of Al composites by combining SLM and *in situ* reaction process.

## 1. Introduction

Laser beam provides a flexible and intense energy source for materials processing [1]. Over the last decade, it is adopted for emerging metallic additive manufacturing applications, in particular selective laser melting (SLM) process which directly produces near net-shape or net-shape parts from metallic powders. The SLM process melts and rapidly solidifies a series of layers on top of each other from powder materials to form complex three-dimensional parts [2–4]. SLM has been mostly used to process metals. For example, Louvis et al. [5] have reported the SLM behaviour of Al alloys or Buchbinder et al. [6] have succeeded to manufacture dense Al alloy parts using a very high power SLM machine. However, as a powder based process, it also provides great opportunity to consolidate second or multiple material particles with metal powders to form novel metal matrix composites (MMCs).

There is a growing research to develop MMCs via SLM process [7]. In particular, SLM is being developed to produce *in situ* composites from powder mixtures in which constituents react together at elevated temperature. In other words, SLM ignites a chemical reaction in the powder mixture besides the melting of metallic powders. The *in situ* interaction is considered to provide benefits in terms of fine and uniform distribution of compounds, inherent interface between reinforcement and matrix, and exothermic energy to facilitate melting and densification. These benefits have been reported in some pioneering research work on SLM of *in situ* MMCs. For instance, Dadbakhsh and Hao [8] have studied the SLM of Al/Fe_2_O_3_ powder mixture for the direct fabrication of *in situ* Al MMCs. The SLM process was found to be capable of activating an exothermic reaction in the powder mixture for the improved material melting and the formation of homogenously distributed fine alumina and Al-Fe(-O) particles, showing a great promise for a novel *in situ* consolidation approach to fabricate advanced Al MMCs and their net-shape components. Despite the research works exploring the SLM feasibility and processability of Al/Fe_2_O_3_ powder mixtures [8, 9], the *in situ* mechanisms with respect to various parameters such as layer thickness require further investigations.

The SLM usually involves rapid solidification (due to the short laser-material interaction time and quick movement of laser after melting). The SLM rapid solidification could modify the microstructure through microstructural refinement, solid solubility extension, and so forth. This can be particularly interesting for Al alloys where the equilibrium solid solubility of many elements is very limited [10, 11]. Therefore, the microstructural characteristic of Al MMC parts made by SLM (with a wide range of non-equilibrium phenomena) differs from those produced by conventional casting methods. These specific microstructural characteristics could lead to advanced material properties for novel net-shape MMCs components made by SLM.

The SLM process activates exothermic *in situ* reaction to form new constituents in the mixture of Al/Fe_2_O_3_ powders and simultaneously leads to rapid solidification manipulating the microstructure. The process parameters can play an important role in between. For example, the powder layer thickness can affect the heat of *in situ* reaction and the solidification (due to change in heat transfer condition) and subsequently the microstructure. In addition, various laser processing parameters such as laser power and scanning speed can influence the material melting and consolidation phenomena. This work has been dedicated to investigate the microstructural outcome of *in situ* reaction in Al/5 wt.%Fe_2_O_3_ powder mixture activated by SLM. Effects of laser power (*P*), scanning speed (*v*), and also layer thickness (*t*) on the microstructural evolution, phase changes, and material microhardness are presented and discussed.

## 2. Materials and Experiments

**Figure 1:**
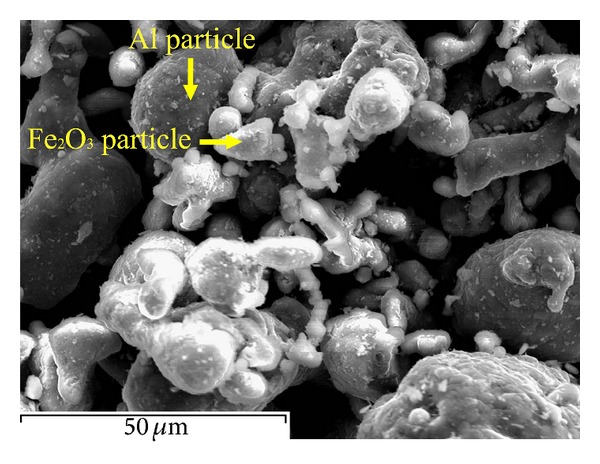
The pure Al/5 wt.%Fe_2_O_3_ particle mixture.

Pure Al (99.7 wt.% purity—mean particle size of 40 *μ*m—Alpoco Ltd., UK) was mixed with 5 wt.%Fe_2_O_3_ powder (mean particle size of 18 *μ*m, Inoxia Ltd., UK), as shown in Figure 1. A SLM machine (Realizer 250, MCP Ltd.) was used to process Al/5 wt.%Fe_2_O_3_ mixture. Argon gas was pumped into the build chamber to keep the O_2_ level below 0.9%. Single layer samples were made on a thick powder bed with a laser power of 51 (W) to demonstrate thick layer thickness properties. Multilayer SLM samples with dimensions of 10 mm × 10 mm × 6 mm were fabricated onto the aluminium substrates using spot size of 0.16 mm, scan line spacing of 0.05 mm, and powder layer thicknesses of 75 *μ*m and 50 *μ*m. A number of multilayer samples were produced in array format with various laser powers in the range of 39–91 W and scanning speeds of 0.14 m/s and 0.5 m/s. Each layer was scanned twice. The scanning was carried out in *x* direction for the first layer and in *y* direction for the next layer, and so on.

The sample *x*-*y* cross-sections (i.e., in the plane of one layer) were cut and polished. To reveal the microstructure, the specimens were chemically etched at room temperature using a solvent composed of 95 mL water, 2.5 mL HNO_3_, 1.5 mL HCl, and 1.0 mL HF. The microstructure was viewed using a Hitachi S-3200N scanning electron microscope (SEM) equipped with an energy dispersive spectrometry (EDS) microprobe system, permitting a comparison study on alternation of chemical composition despite slight inaccuracy which might occur due to etching.

Phase identification of product was carried out using a Bruker D8 Advance X-ray diffractometer (XRD) with coupled 2Theta/Theta scan type and Cu-K*α*1 radiation (wavelength 0.15406 nm). The phases were identified using the machine software (DIFFRAC.SUITE), allowing the identification of low percentage of secondary phases (i.e., weak peaks) with a high accuracy. The hardness was measured using Vickers microhardness test (100 g load was applied for 30 s) from the average of at least 5 hardness readings in conjunction with a Future-Tech Microhardness Tester FM testing machine.

## 3. Results

### 3.1. Hardness Properties

**Figure 2:**
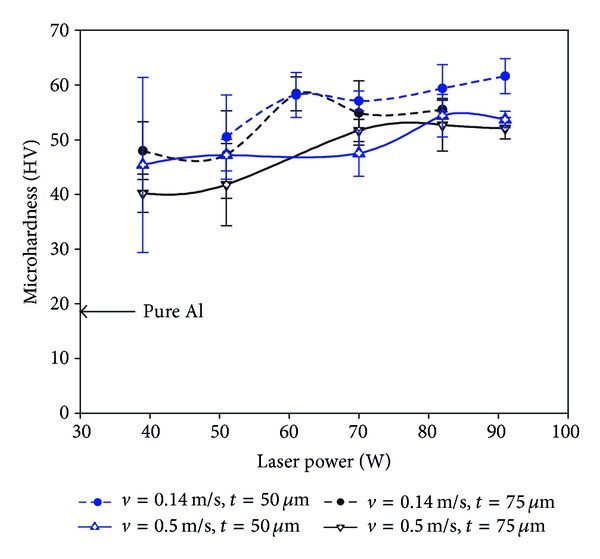
Microhardness of the multilayer parts fabricated with layer thicknesses of 50 *μ*m and 75 *μ*m and scanning speeds of 0.14 m/s and 0.5 m/s versus laser power.

Microhardness tests were carried out to investigate the evolution of microstructural hardness with various laser powers when the multilayer parts were manufactured with layer thicknesses of 50 *μ*m and 75 *μ*m at two different scanning speeds (*v* ~ 0.14 m/s and 0.5 m/s). As shown in Figure 2, hardness usually increases with laser power, but it may reach a maximum value. For example, the hardness in the curve of *v* = 0.5 m/s and *t* = 75 *μ*m increases up to about 82 W reaching a maximum value of about 53 HV and then it starts to slightly decrease with further increase of laser power. Scanning speed also affects the hardness; that is, lower scanning speed leads to higher hardness (cf. hardness curves with different scanning speeds). Moreover, thinner layer thickness of 50 *μ*m may only have a very slight effect to enhance hardness. The highest hardness obtained in this SLM operation window belongs to the part manufactured by *P* = 91 W, *v* = 0.14 m/s, and *t* = 50 *μ*m, which is about three times hardness value of pure aluminium (no conventionally manufactured Al composite with the comparable reinforcement characteristics was found in the literature to be used as a basis of the comparison) [12].

### 3.2. Microstructural Characterisation and Phase Identification Using XRD

#### 3.2.1. Single Layer Specimens Made by SLM on a Thick Powder Bed

**Figure 3:**
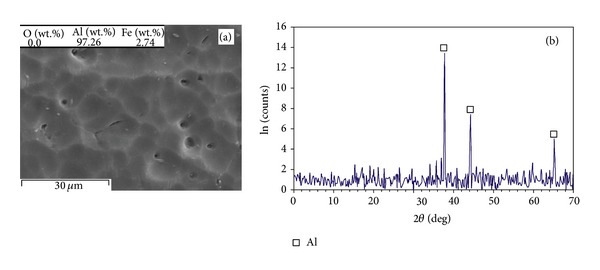
(a) Typical SEM microstructural view showing a granular structure (overall chemical composition was acquired by EDS) and (b) XRD pattern of the part fabricated on a thick powder bed using *P* = 51 W and *v* = 0.14 m/s.

The single layer specimens manufactured from a thick powder bed can represent a very large thickness for comparison study of various SLM layer thicknesses.  Figure 3(a) exhibits a granular and integrated matrix (grain size ~5–15 *μ*m). The EDS analysis implies that the oxygen has been efficiently removed from the Al matrix.  Figure 3(b) shows the XRD pattern of the part, demonstrating that the matrix is a single Al phase.

#### 3.2.2. Multilayer Parts with Layer Thickness of 75 *μ*m

**Figure 4:**
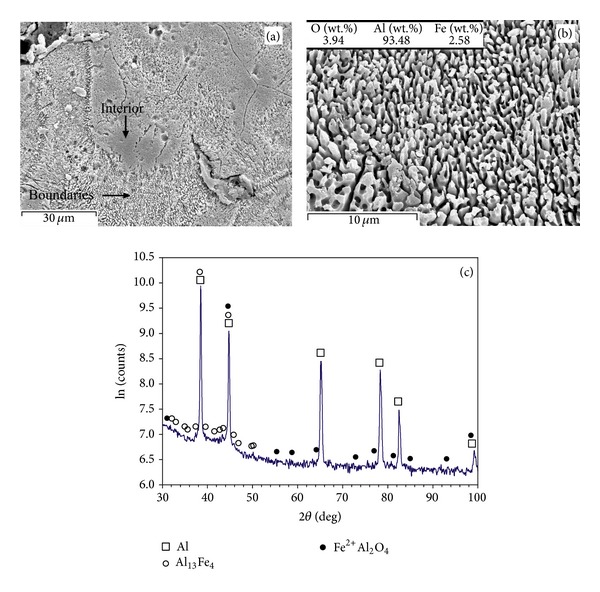
(a) Overall view of microstructure, (b) higher magnification of coralline-like and fuzzy appearance of boundaries in the previous image, and (c) XRD pattern of the SLM part fabricated when *P* = 39 W, *v* = 0.5 m/s, and *t* = 75 *μ*m.

**Figure 5:**
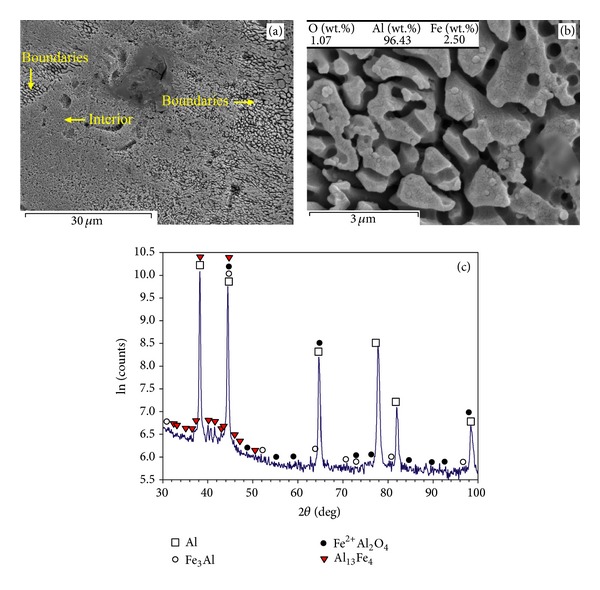
(a) Overall view of microstructure, (b) boundaries seen in the previous image, and (c) XRD pattern of the part fabricated when *P* = 39 W, *v* = 0.14 m/s, and *t* = 75 *μ*m.

Figures 4 and 5 show the SEM microscopic graphs of the multilayer parts (built with *t* = 75 *μ*m and *P* = 39 W) under different laser speeds of *v* = 0.5 m/s and *v* = 0.14 m/s, respectively.  Figure 4(a) exhibits grains (smooth, with a size of about 30–50 *μ*m) surrounded by thick boundaries (appearing with a fuzzy appearance). The fuzzy appearance of boundaries may look like a coralline-like feature (the coralline tips are ~0.5 *μ*m), as shown in Figure 4(b). The XRD results (Figure 4(c)) suggest that the part is composed of combinations such as Al, Fe^2+^Al_2_O_4_, and Al_13_Fe_4_ (weak peaks of Al_13_Fe_4_ indicate low content of this intermetallic). The lower scanning speed (*v* = 0.14 m/s) leads to almost the same overall microstructure (Figure 5(a)), though the thick boundaries appear coarser (the coralline tips are in an approximate range of 1-2 *μ*m), as better demonstrated in Figure 5(b). The X-ray pattern (Figure 5(c)) showed Al and Al-Fe intermetallics such as Fe_3_Al and Al_13_Fe_4_ intermetallics and Al-Fe(-O) combinations such as Fe^2+^Al_2_O_4_ (iron aluminium oxide), while apparent presence of Al_2_O_3_ was not identified.

**Figure 6:**
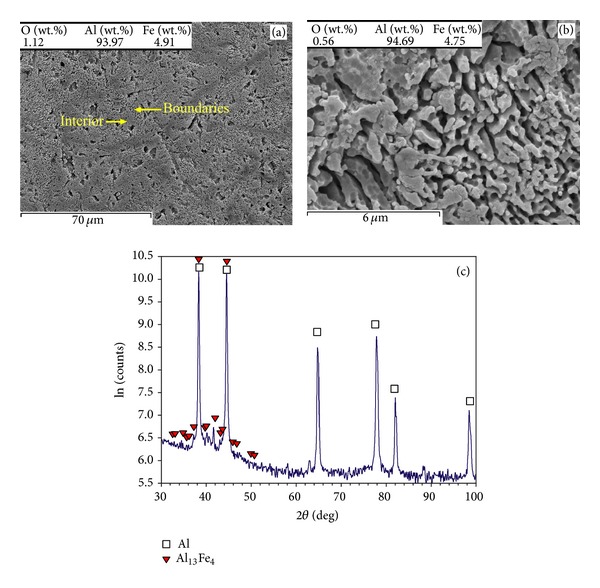
(a) Microstructure, (b) the interior seen in the previous image, and (c) XRD pattern of the part fabricated when *P* = 61 W, *v* = 0.14 m/s, and *t* = 75 *μ*m.

**Figure 7:**
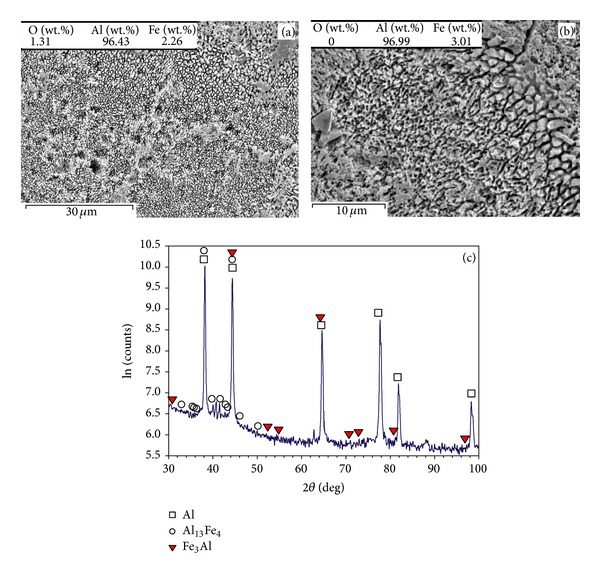
(a) Overall appearance of the grain structure, (b) higher magnification of coralline-like and fuzzy appearance in the previous image, and (c) XRD pattern of the part fabricated when *P* = 82 W, *v* = 0.5 m/s, and *t* = 75 *μ*m.

The part fabricated by *P* = 61 W, *v* = 0.14 m/s, and *t* = 75 *μ*m contains a porous and coralline-like feature propagated throughout the sample, while primary boundaries in narrow lines are recognisable (Figures 6(a) and 6(b)). The chemical composition (composing of Al and Fe with negligible content of oxygen) is in confirmation with the XRD results demonstrating Al and Al_13_Fe_4_ (Figure 6(c)). However, higher scanning speed (*v* = 0.5 m/s) even in higher laser power (*P* = 82 W) and scanning speed (*v* = 0.5 m/s) leads to the finer coralline-like features (in submicron sizes), as shown in Figures 7(a) and 7(b). These features are from Al_13_Fe_4_ and Fe_3_Al with negligible amount of oxygen (Figures 7(b) and 7(c)).

#### 3.2.3. Multilayer Parts with Layer Thickness of 50 *μ*m

**Figure 8:**
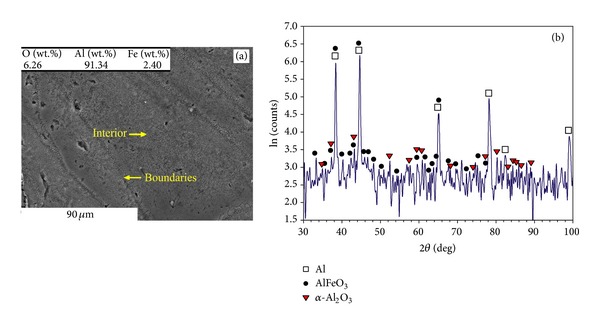
(a) Microstructure and (b) XRD pattern of the part made by *P* = 91 W, *v* = 0.5 m/s, and *t* = 50 *μ*m.

**Figure 9:**
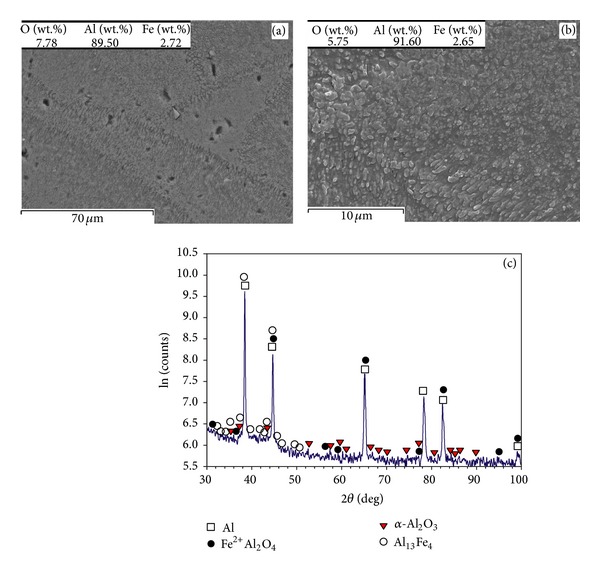
(a) Grain structure, (b) fine particles formed inside grains and boundaries (closeup of the previous image), and (c) XRD pattern of the part made by *P* = 91 W, *v* = 0.14 m/s, and *t* = 50 *μ*m.

As shown in Figures 8(a) and 8(b), the microstructure of the parts fabricated at *P* = 91 W, *v* = 0.5 m/s, and *t* = 50 *μ*m contains grain with a semicoralline-like feature from Al, iron aluminium oxides like AlFeO_3_, and Al oxides like *α*-Al_2_O_3_ (alumina). At low scanning speed (*v* = 0.14 m/s), the homogeneous distribution of very fine particles (in nanosizes and submicron scales) within polygon grains is observed (Figures 9(a) and 9(b)). The coarser particles (~0.6–0.8 *μ*m) form boundaries, while finer particles (~0.2-0.3 *μ*m) are embedded inside grains (Figure 9(b)). The XRD pattern (Figure 9(c)) corresponds to Al, Fe^2+^Al_2_O_4_ (iron aluminium oxide), and *α*-Al_2_O_3_ (alumina). The diffraction peaks for Al_13_Fe_4_ are also detected, though the intensity is comparatively weak.

By the inspection of Figures 3–9, the effect of layer thickness and laser parameters on the microstructural characteristics and identified phases can be summarised, as shown in Table 1. This will be discussed in Section 4.

## 4. Discussion

### 4.1. *In Situ* Interaction Initiation

Through mechanical blending, the pure aluminium (Al) and iron oxide (Fe_2_O_3_) powders can be uniformly distributed and fully mixed together, whilst the particles tend to adhere randomly as seen from Figure 1. This adherence is very helpful for SLM process to activate the *in situ* reaction between Al and Fe_2_O_3_ particles. In fact, in an initial stage of laser-material interaction, the energy is absorbed in a narrow layer of individual powder particles, leading to a high temperature of the surface of particles during the interaction [13, 14]. The melting/exothermic reaction starts from these interfaces between Al and iron oxide interface and the thermite reaction progresses regarding the following stoichiometric equation:

(1) $$\begin{array}{c} 8\text{Al}+3{\text{Fe}}_{2}{\text{O}}_{3}⟶2{\text{Fe}}_{3}\text{Al}+3{\text{Al}}_{2}{\text{O}}_{3}+\text{heat} \end{array}$$

The final phases, Al_2_O_3_ and Fe_3_Al intermetallics, are formed by an *in situ* chemical reaction in which Al reduces the iron oxide [15, 16]. The extra heat from *in situ* reaction facilitates melting during SLM process. The mechanism of reaction involves reducing Fe_2_O_3_ to Fe_3_O_4_ and FeO by releasing the oxygen and Al oxidation, interaction of Al with iron oxides, and formation of phases such as iron-aluminium-oxide, alumina, iron, and Al-Fe intermetallics, depending on reaction temperature and Fe_2_O_3_/Al ratios [17, 18].

### 4.2. Effect of Layer Thickness, Laser Power, and Scanning Speed on Oxide Formation

The oxygen content as the main factor for quantity of oxides is significantly altered according to the parameters used to manufacture parts. This can be attributed to relation of oxide formation with temperature. The increased reaction temperature (due to higher laser power) and inert atmosphere lead to the decomposition of Fe_2_O_3_ into FeO and Fe and oxygen which escapes from the specimen [18]. In a parallel manner, the vaporisation of thin oxide films to fume can occur on the top of the Al molten pool in high temperatures of laser/material interaction [5]. The released oxygen and oxide film fume can be carried away by the inert gas flowing over the bed [19]. The Marangoni forces can contribute to this oxygen removal by stirring and breaking oxides [5]. On this basis, higher laser power and lower scanning speed (leading to higher temperatures) should contribute to oxygen removal by intensifying the melting flow, decomposition, and evaporation [5, 18]. For example, low scanning speed of 0.14 m/s in Figure 5 results in lower comparative oxygen than that of Figure 4 (*v* = 0.5 m/s).

As observed from Figures 8 and 9 (*t* = 50 *μ*m) to Figures 4–7 (*t* = 75 *μ*m) and Figure 3 (thick powder bed), the higher oxygen appears in lower layer thickness. This is because the thicker layer thickness reduces the oxygen by enhancing temperature and stirring (since a thicker layer reduces the cooling rate, activates more exothermic reaction and produces a larger melting pool). The higher oxygen contributes to the formation of hard oxides such as aluminium oxide (Al_2_O_3_) or iron aluminium oxides (like Fe^2+^Al_2_O_4_ and AlFeO_3_), reinforcing the matrix.

### 4.3. Microstructures, Phases, and Microhardness

The single Al phase granular feature (Figure 3), formed in the SLM specimen built on a thick Al/Fe_2_O_3_ powder bed, can be attributed to enhanced oxygen removal at large layer thickness. This leads to an Al matrix saturated by Fe (SLM rapid solidification from high temperatures assists saturation of Al by other elements [11, 20]). This contributes to strengthening by solution hardening.

The multilayer parts exhibit higher microhardness than that of the conventional pure aluminium (Figure 2). The lowest hardness belongs to Figure 4 in which fuzzy and coralline-like boundaries have just started to develop into interior. These phases are Al-Fe intermetallics and Al(-Fe) oxides, demanded for hardening/strengthening purposes (e.g., Al oxide has been used as reinforcement in Al alloys [21] or Al_13_Fe_4_ has been recognised as major hardening phase in surface hardening of Al by Fe-ion implantation [22]). Therefore, they start to enhance the hardness of Al matrix compared to pure Al. Considering the detected combinations, Al_13_Fe_4_ (equilibrium) is a result of transition from Fe_3_Al (the ordinary production of (1)) due to excessive Al [23]. Also, Fe^2+^Al_2_O_4_ has been formed due to its lower activation energy than that of Al_2_O_3_ [15], though it tends to transform into Al_2_O_3_ and Al-Fe intermetallics [18].

The average particle size of Al powders (about 40 *μ*m) results in a similar range of grain size in Figure 4(a). Higher laser power of 82 W develops coralline-like feature (Figure 7) from ultrafine intermetallics such as Al_13_Fe_4_ and Fe_3_Al (Figure 7(c)). This is accompanied with removing oxides from matrix and lowering the oxygen content (Figures 7(b) and 7(c)). Despite the absence of oxides, the fine and hard Al-Fe intermetallics enhance the hardness (see the corresponding microhardness curve in Figure 2).

Both Figures 4 and 5 contain coralline-like features in boundaries, however the corallines in Figure 5 are slightly further developed due to lower scanning speed. In fact, lower laser speed enhances the laser input and decreases the cooling rate leading to coarser corallines (the coralline tips are ~1-2 *μ*m in Figure 5, but ~0.5 *μ*m in Figure 4). Despite the coarsening effect, enhancing the melting (by increasing laser input) results in further propagation of these Al-Fe intermetallics into interior, a more uniform microstructure, and a higher hardness. This completes in higher laser powers (Figure 6), leading to the highest hardness of the relevant curve (treated at *P* = 61 W, *v* = 0.14 m/s, and *t* = 75 *μ*m—Figure 2). After this, the hardness may fall despite the bonding improvement, perhaps due to the excessive coarsening or formation of other softer *in situ* products.

Decreasing the layer thickness of the parts to 50 *μ*m changes the sharp-pointed coralline-like appearance of microstructure (Figure 7) into semicorallines and particles (Figures 8 and 9). The oxygen content is considerably higher than what was observed in higher layer thickness (e.g., cf. Figures 8 to 7), increasing the oxide particles.

Reducing the laser speed in conjunction with lower layer thickness of 50 *μ*m (Figures 9(a) and 9(b)) leads to uniform distribution of very fine particles (in nanosize and submicron scale) with strong bonding within the matrix. This results in the highest hardness (Figure 2). Formation of these reinforcing particles can be attributed to the used parameters. In fact, the lower layer thickness intensifies fragmentation of corallines (by remelting the ex-layers), refines the microstructure (by increasing the cooling rate), and increases the oxide particles (by restricting the oxygen removal). Besides, the lower laser speed contributes to making particles by assisting the oxide fragmentation.

### 4.4. *In Situ* Combined SLM Phenomena

The Al-Fe equilibrium binary diagram shows a eutectic point around 1.8 wt.% iron which is composed of Al and Al_3_Fe (Al_13_Fe_4_) [23, 24]. The eutectics usually tend to form with a lath-like morphology, but, here, the rapid solidification restricts their growth. Thus, these incomplete Al-Fe lath-like eutectics appear with a coralline-like morphology in areas with low oxygen content (e.g., Figure 7(b)). The existence of coralline-like phases has rarely been reported by other researchers. For example, Wong et al. [20] observed that the eutectic silicon in Al-Si alloys was strongly modified during laser treatment, in such a manner that silicon crystal in eutectic was changed morphologically from lath-like into coralline-like.

As summarised in Table 1, the SLM of single layer specimens on a thick powder bed forms an Al granular feature saturated by Fe. When the 75 *μ*m layer thickness is used, low laser energies form the coralline-like phases (being in fact rapidly solidified Al-Fe eutectics), though Al oxide (mainly in the form of particles) may also be found from surface oxide fragmentations or *in situ* products. Higher laser power develops the *in situ* reaction leading to incorporation of Al into Fe^2+^Al_2_O_4_ and transforming it into Fe_3_Al and then equilibrium Al_13_Fe_4_ (as much as possible depending on the condition). Simultaneously, oxygen removal is adequate to mitigate the oxides, leaving mainly Al and Al-Fe intermetallics. Thus, coralline-like features propagate all over the samples in these conditions. A decrease in layer thickness into 50 *μ*m suppresses the oxygen removal and increases the stable and particulate Al_2_O_3_. It also increases the solidification rate which decreases the transformation of iron aluminium oxides (like Fe^2+^Al_2_O_4_ and AlFeO_3_) into intermetallics. The lower scanning speed may increase the fragmentation of oxide layers. This collaborates with the ex-layer remelting which breaks the corallines formed in previous layers. Consequently, coralline-like appearance may disappear and particulate reinforced Al matrix may be revealed.

## 5. Conclusions

- *In situ* reaction was successfully activated by using SLM process in a powder mixture of Al/5 wt.%Fe_2_O_3_. The microstructural evolution of SLM parts was investigated in the dependence of various parameters such as powder, layer thickness, laser power and scanning speed.
- Thicker layers lead to more efficient oxygen removal during SLM. This can be considered as the main reason for the large microstructural differences with changing the layer thickness.
- The microstructure after SLM of a thick powder bed, 75 *μ*m layer thickness, and 50 *μ*m layer thickness appeared with dominantly granular, coralline-like (being in fact rapidly laser solidified Al-Fe eutectics), and coralline-particulate and particulate features, respectively. These microstructures represent mainly pure Al, Al-Fe intermetallics (such as Fe_3_Al and Al_13_Fe_4_), and Al(-Fe)-O oxides (such as Al_2_O_3_, Fe^2+^Al_2_O_4_, and AlFeO_3_) after rapid solidification.
- The earliest *in situ* products are phases such as Fe_3_Al and Al(-Fe)-O oxides (e.g., Fe^2+^Al_2_O_4_), but they tend to transform into more stable combinations such as Al_13_Fe_4_ and *α*-Al_2_O_3_.
- Higher laser power also contributes to more efficient oxygen removal during SLM. Higher scanning speed leads to finer Al-Fe coralline-like features, and it may restrict the *in situ* progress and transformation of products.
- A very promising result (useful to manufacture Al matrix composites) was achieved by using low layer thickness, high laser power, and low scanning speed. This led to well-bonded and uniform distribution of fine, hard, and stable particles in Al matrix and consequently resulted in the highest comparative microhardness.

## Figures and Tables

**Table 1:** Summary of microstructural evolution in the dependence of layer thickness, laser power, and scanning speed.

	Thick powder bed	*t* = 75 *μ*m	*t* = 50 *μ*m
Morphology (appearance)	Single Al phase-granular structure	Predominantly coralline-like	Semicoralline-like/particulate

Oxygen/oxide content	Least oxygen/oxide	Medium oxygen/oxide	Highest oxygen/oxide

Predominant phases	Al	Al, Al-Fe intermetallics such as Al_13_Fe_4_ and Fe_3_Al; some oxides (like Fe^2+^Al_2_O_4_) may also be detected	Al, aluminium oxide (Al_2_O_3_) plus iron aluminium oxides (e.g. Fe^2+^Al_2_O_4_ and AlFeO_3_), Al-Fe intermetallics such as Al_13_Fe_4_

Increasing laser power	N/A	Development of corallines (mainly Al-Fe intermetallics) and oxide particles from primary Al/Fe_2_O_3_ interface—reduction in oxygen/oxide	N/A

Lowering scanning speed	N/A	Coarsening of corallines—reduction in oxygen/oxide	Better dispersion and placement of secondary phases
